# RACIAL-ETHNIC DIFFERENCES IN NURSING HOME QUALITY OF LIFE FOR ALZHEIMER’S DISEASE AND DEMENTIA RESIDENTS

**DOI:** 10.1093/geroni/igz038.1527

**Published:** 2019-11-08

**Authors:** Tetyana P Shippee, Stephanie Jarosek, Xuanzi Qin, Mark Woodhouse

**Affiliations:** 1 University of Minnesota, Minneapolis, Minnesota, United States; 2 U of MN, Minneapolis, Minnesota, United States; 3 School of Public Health, University of Minnesota, Minneapolis, Minnesota, United States

## Abstract

Nursing homes (NHs) are often racially segregated, and minority residents admitted to NHs usually have more advanced stages of dementia at the time of admission than their white counterparts, with different care needs. Previous work has shown that racial disparities in NH quality of life (QoL) were partially due to different case mix of white and minority residents; it is unclear if disparities persist when comparing residents with similar ADRD diagnoses. The 2011-2015 Minnesota Resident Quality of Life and Satisfaction with Care Survey data contain in-person resident responses from a random sample of residents of all Medicare/Medicaid certified NHs in the state, about 40% of whom have AD/ADRD. These data were linked to the Minimum Data Set (MDS) and facility characteristics data. The population consists of 25,039 White, 580 Black, 94 Hispanic, 229 Native Americans, and 99 Asian/Pacific Islander NH residents with ADRD residing in 376 NHs. Racial/ethnic minority residents reported significantly lower QoL scores compared to their white counterparts, with the largest disparities in the food and relationships domains. We adjusted for resident (age, marital status, education, sex, length of stay, anxiety/mood disorder, activities of daily living scores) and facility characteristics (proportion of minority residents, ownership, urban vs rural, size, and occupancy ratio) using a multivariate random intercept model. After adjustment, significant differences remained in total QoL score and several QoL domains for Black, Asian and Hispanic residents (no significant differences for Native American residents). Practice guidelines should consider different care needs of racial/ethnic minority NH residents with ADRD.

